# Medical Quackery

**Published:** 1846-01-01

**Authors:** WM. Treat


### Medical Quackery.

Communicated for the Buffalo Medical Journal, by WM. TREAT, M. D.

At a time when square feet of parchment, called diplomas, a term now nearly obsolete, were exhibited to solve the inquiry, "what is quackery," it could thus be easily answered. The *spirit* of the inquiry has not so much changed since then, we apprehend, as this answer has lost its significancy. With this lost, however, the query takes new form, and with this form comes increased difficulty in framing a reply. Yet, for an answer the intelligent of community still call, as aid in determining who is the truly scientific physician, deserving in all respects their confidence and trust. That this difficulty is increased much, very much, by the profession itself, and with it the prevalence of quackery, we hesitate not to say; and that, in the first place, by the reception of students, deficient, often sadly deficient in an elementary and preparatory education; students serving as economical office attendants, and from their number giving consequence to the physician, like retinue to royalty. This system of student-making advances to physician-making, when acted upon by our medical institutions in their strife for numbers, as upon these, the fame of the institution is based, and the result sought, is attained, viz: pecuniary gain to its Professors. But the evil rests not here; to the show of numbers in catalogue, a large list of graduates, diplomated in each half year, is superadded, and these embodied in annual announcements, serve as home appeals to the feverish hopes of the aspirant for diplomated honors, since from these lists he is led to infer the *facile* of graduation, a consummation, too often, of all ambition.

With such evils attendant upon the system of granting diplomas, what can be said of another evil which is fostered by States, by Counties, and by Counties' Deputies, which, like recruiting officers, drum up students, be they well or illy qualified, and enlist them under the banner of those presumed in the true faith, and this by nominal examinations, made perhaps during the hurry of professional business; and, be the applicants few or many, they are speedily passed upon, accepted, duly enrolled, and sent abroad under official sign manuals to be jeered at for their incompetency, often by those very individuals who have overlooked their individual responsibility to the profession, and to the public, when called upon to decide in their collective capacity.

The results of such proceedings are daily apparent. To be a student in medicine has come to be no evidence of revolt from the dominions of duncedom—to be a graduate, no evidence of freedom from quackery, for such has been the rapid increase the past few years, of such illy qualified practitioners, the *profession* is nearly lost, like good wheat, by being mixed in the mass of dwarfed kernels, and excess of chaff; or, for want of an institution to serve as a refiner's fire to separate, by assay, their real value; in other words to ascertain, not who has the greatest, but the smallest modicum of knowledge with which he may be tolerated as a practitioner; for this, and this only, is all that we can reasonably expect diplomas to set forth, unless they differ in kind, as is the case with academic degrees.

In passing over the length and breadth of the land, where shall the mind rest upon such an institution? That each set forth claims—that many have merit, we do not question. But what has the community, the profession, even, as surety that their professors are capable to teach?—Have they passed any public ordeal, any professional ordeal of examination? Have they earned a reputation by experience in practice of the profession? Have they walked the rounds of a hospital, even for appearance's sake, to say nothing of a fitting time? Have they moral qualifications requisite to set aside all personal friendships, or even to refuse all expected pecuniary reward of present graduate's, fee, or remote, by show of the large list sent forth in the annual announcement? Let the most partial graduates of each institution answer. But diplomas from foreign institutions are sometimes offered in testimony of ability as teachers and practitioners. Of the mode in which they may be obtained in Great Britain, we need not detail after speaking so freely of those in our own country; for from that country we have the models from which our institutions are framed, omitting some of their most offensive measures, viz: that power conferred upon the Archbishop of Canterbury to bestow medical degrees at pleasure;—the bestowal of medical diplomas upon those who reside at Cambridge or Oxford College two years, on the payment of a few pounds sterling, and on condition they practice out of the larger towns. Nor are we aware that in this country competition has ever been so active as that at "Marischal College" (in Aberdeen, Scotland,) with its rival 'Kings,' where the degrees in medicine were offered to academic students gratis! For this reason, in passing review, we would add, it is, that the profession should, and do generally feel called upon to refuse fellowship with all those who present diplomas from Europe, until they shall have subjected themselves to an examination in this country, aside from the uncertainty that the holder may not be the person for whom the diploma was originally designed. To this examination, therefore, no physician who has the reputation of his profession and the weal of the community at heart, can reasonably object, be his standing abroad what it may. If qualified, he certainly can suffer nothing in the trial, and may thus certainly be helped onward unincumbered by that suspicion which frequent deceptions from such sources, now naturally attaches to all.

But to return:—the lamentable prevalence of quackery, we repeat, originates in a great degree in the profession. The prevalent opinion, naturally enough formed, is, that students are fitted for practice by the instructions of those in whose offices they are found, thus judging, as of public and private teaching in any other educational department. Herein that opinion is ignorant of the custom which obtains among the profession, and is almost universal, viz: of simply placing books before the student, however illy he may be prepared to analyze them; and the time of study—three years—is suffered to pass without farther care on the physician's part, often no recitations being had, nor oral instruction given.

At Medical Colleges, also, this is true of recitations, for "taking the quiz," cannot be considered a recitation—and if the student have memory sufficient, or has been "crammed," as the phrase is, he passes an examination, which, in many institutions are completed in an hour! In such a state of things the answer to the query, "In what consists Medical quackery?" must necessarily be difficult to frame. But we would not leave the subject without an attempt; we answer then, it is a pandering to the gullibility of human nature; an issue of base coin so tempered as to deceive the inexperienced by its sound, its jingle.

Who then is the quack? He who moves in words or manner, in person or by deputy, to convey an impression of merit beyond the consciousness of his intrinsic worth. Specifically, he who by horse, or on foot, runs to and fro pretending to be busiest of the profession. He who stoops to self-commendation, directly or indirectly, vaunting of opportunity enjoyed for study, gossips of cases, of want of rest, and sleepless nights, or suffers this to be said of him by kith or kin as part of machinery working to increase of capital, or makes a partnership affair by jackall appliances, preying upon spoils filched by the one who stoops where the other partner could not. He who flourishes in the use of one of the most difficult of all instruments, the stethoscope, pronounces off-hand in cases in which all sense, including common sense, knows repeated and patient examinations are requisite: who makes the smallest operations *capital* ones, builds upon them, magnifies them, in short, does what a conscientious man could not. Such, is the quack. Diplomas of parchment cannot make him otherwise, for the stamp upon metal makes not the coin golden: *brass* is known to take the lettered impression equally well, and with the mass may pass undetected.

Thus it will be seen that the quack *without*, may be found often in grade even with the well-to-do *in* the profession. Indeed it is very difficult to tell where the 'regular' and 'irregular' touch palms, since the veriest charlatan may rise in mental endowment above the obtuse or torpid licentiate, pillowed upon his scroll of parchment. The remedy for this condition of things we have somewhat foreshadowed and may not now speak, warned as we are, that if necessity demands free utterance, knowing neither metes nor bounds, such is not the case with the limited pages of this Journal. We have felt driven to speak with much stringency, more, perhaps, than the existing state of the profession to the kindly reader would warrant. Were we not addressing the profession we should say much in mitigation, much laudatory of the honorable course its members, many of them, have pursued in these portentous times. But while we reprove the evil, we trust that what we have written will not be construed into a want of perception of the good, but on the contrary urge to a greater emulation for its attainment.

BUFFALO, June, 1845.

---

REMARKS BY THE EDITOR.—The writer of the foregoing has intended to satirize defects and malversations appertaining to medical education and professional conduct, which, however mortifying the acknowledgment may be, it must be confessed prevail to a lamentable extent. The ranks of the profession are crowded with members who are sadly deficient in preliminary and medical acquirements, as well as in those enlarged views and elevated sentiments, which are associated with the duties and relations of the position which the practitioner, from the nature of his calling, should occupy. There is, unfortunately, no occasion to adduce arguments or illustrations to confirm the correctness of this assertion. It is, alas, a fact but too apparent to every competent observer. But the great question which concerns us in view of this fact, our correspondent has deferred. This question is, *what is the remedy?* Is there any method by which present evils can be corrected, and the improvement so much to be desired, effected? This is an inquiry which it behooves us to ponder, and which we doubt not occupies at the present moment the earnest thoughts of many who have the honor and usefulness of the profession at heart. What is to be done? We cannot look to legislation. We cannot depend on the public in any mode to originate a movement toward a better condition of things. Our only dependence, the dependence of the profession, in other words, is upon itself—upon ourselves. No other mode at present suggests itself to our minds, but to unite in an organization, and originate a tribunal of some sort, competent to take cognizance of the qualifications necessary to constitute an approved member of the medical profession. This tribunal should secure uniformity of qualification throughout our country. It is needless to add, that it should be independent of all extrinsic influences, and ever vigilant to protect the character of the profession. To form such a tribunal, and to give to it the needed moral force, no powers delegated by legislation are necessary. If sustained by the united voice of the well-informed and honorable members of the profession, the potency of the public *will* would follow, to give it efficacy and permanency. Diplomas and Licenses, which have degenerated into mere nominal insignia, would be compelled to become subordinate to its decisions. The limits of the profession would be defined, and each member would to some degree, represent its true character. It is far otherwise now! We throw out these remarks as suggestives to reflection. We may possibly continue the subject in another number. It is a subject immensely important, and one which at the present time should not fail to occupy the thoughts of the profession. A national convention, it is hoped, is soon to take place, when the incipient steps of some method of reform may be taken. We give the ideas which recommend themselves to our own judgment, but we are not wedded to any particular plan, and shall be glad to have an opportunity of comparing our views with those of othets.

